# May osteoarticular infections be influenced by vitamin D status? An observational study on selected patients

**DOI:** 10.1186/s12891-015-0648-5

**Published:** 2015-08-05

**Authors:** Valentina Signori, Carlo L. Romanò, Elena De Vecchi, Roberto Mattina, Lorenzo Drago

**Affiliations:** Laboratory of Clinical Chemistry and Microbiology, IRCCS Galeazzi Orthopedic Institute, Via R. Galeazzi 4, 20161 Milan, Italy; Center for Reconstructive Surgery of Osteoarticular Infections (C.R.I.O.), IRCCS Galeazzi Orthopedic Institute, 20161 Milan, Italy; Department of Biomedical, Surgical and Dental Sciences, University of Milan, 20122 Milan, Italy; Department of Biomedical Science for Health, University of Milan, 20133 Milan, Italy

## Abstract

**Background:**

Vitamin D deficiency has been associated with a high number of health outcomes, and its role on the immune system has been deeply investigated in recent years, although poor data are still available on vitamin D status in orthopedic infections including those of prosthetic implants.

**Methods:**

We focused on preoperative values of 25(OH)D in selected groups of patients with septic (Group A) or aseptic (Group B) prosthetic loosening, infective bone disease such as septic arthritis and osteomyelitis (Group C) and other orthopedic pathologies (Group D) to evaluate differences in the vitamin D status.

**Results:**

A high prevalence of vitamin D deficiency was recorded among the study population (16.5 ± 5.4 ng/mL, mean ± SD). Interestingly, all patients with an infection presented a higher 25(OH)D concentration (17.7 ± 5.3 ng/mL) in respect to uninfected ones (15.1 ± 5.6 ng/mL). Significantly higher levels of 25(OH)D were observed in patients with prosthetic joint infection (18.5 ± 6.5 ng/mL), when compared with those presenting an aseptic loosening (13.6 ± 9.4 ng/mL).

**Conclusions:**

Deficiency in vitamin D levels have been found in orthopaedic patients. Prosthetic joint infections seems to be associated to higher values of vitamin D in respect to other bone infections or to other orthopaedic conditions requiring surgery.

More studies are needed to improve the knowledge on vitamin D status in these patients and to better clarify the role of vitamin D in relation to osteoarticular infections.

## Background

Vitamin D plays several roles in the body, influencing bone health as well as serum calcium and phosphate levels, innate immunity function, cell proliferation, differentiation and apoptosis. The ability to synthesize vitamin D in the skin lessens with aging [1] and suboptimal levels may negatively impact on calcium metabolism, osteoblast activity, matrix ossification and bone density, [2] and even on the immune system [3]. Vitamin D circulating form, 25(OH)D, presents highly variable levels in the general population, and varies with geographical latitude, solar exposure and age, acting as an important confounder in the quantification of the net effects of vitamin D.

Hospitalized patients are often vitamin D deficient and nevertheless, vitamin D status is a critical factor for maintaining musculoskeletal health and, therefore, it requires particular consideration in patients undergoing orthopedic surgery, [4, 5] even if the role of vitamin D on muscular and physical functions after primary arthroplasty is not clear.

This is potentially important because vitamin D deficiency can be easily and safely corrected by 6 weeks oral vitamin D supplementation [6, 7]. As a consequence, a screening for vitamin D deficiency in individuals at risk, as well as oral vitamin D supplementation before orthopedic surgery could be advisable, once demonstrated an association between low vitamin D levels and poorer functional outcomes.

Given its influence on the immune system and the inflammatory cascade, vitamin D may have in future an important role in prevention and treatment of infection. Concentrations of vitamin D have been found to be inversely correlated with risk of infections by multiple epidemiological studies in adults and children that have demonstrated that vitamin D deficiency is associated with increased risk and greater severity of infection, in particular regarding the respiratory tract [8, 9].

However, data on vitamin D in orthopedic patients in relation to infections are rather scarce, so the purpose of the present study was to determine vitamin D serum concentration in patients undergoing orthopedic surgery for prosthetic or bone tissue infections, and to compare vitamin D levels between these patients and non-infected ones.

## Methods

We retrospectively collected data from patients scheduled for orthopedic surgery at the Centre for Reconstructive Surgery of Osteoarticular Infections (C.R.I.O.), of Galeazzi Orthopedic Institute in Milan, during the period from February to September 2013.

Patients presented prosthetic failure (aseptic loosening or Prosthetic Joint Infection – (PJI)), osteoarticular infections (septic arthritis or osteomyelitis) or other osteoarticular pathologies (fractures, arthrosis, ligament reconstruction).

Preoperative demographic characteristics were registered for all patients, including age, BMI, medical and drug history (including vitamin D and calcium supplementation) and co-morbid status.

We selected patients with age ranging between 55 and 65 years, excluding those receiving vitamin D supplementation, or with obesity, osteoporosis, autoimmune diseases, diabetes, hepatitis, renal failure and serious cardiopulmonary disease, in order to obtain an homogenous study group and to minimize statistical multivariable adjustment needs.

The study was performed in accordance with the guidelines of the Helsinki Declaration for biomedical research involving human subjects, and the study was approved by the Review Board of the Galeazzi Orthopedic Institute. Written informed consent for participation in the study was obtained from all patients.

Prosthetic loosening was diagnosed on preoperative radiography and infection was diagnosed when the patient presented elevated erythrocyte sedimentation rate (ESR) and C reactive protein (CRP) concentration, elevated synovial leukocyte count, presence of purulence in the affected part and isolation of a microorganism in intraoperative tissue cultures or prosthetic implants, according to Zmistowski et al. [10].

Patients were divided into four groups: patients undergoing prosthetic revision as a consequence of PJI (group A) or for aseptic loosening (group B), patients with osteoarticular infections but without any prosthetic implant (group C) and patients with other osteoarticular pathologies (group D).

Blood samples were obtained at the preoperative visit about 30 days before surgery. Samples were centrifuged (3000 *g*, 4 °C, 10 min), and serum was stored at −80 °C until analysis.

Serum 25(OH)D levels were measured using an automated CMIA assay (25-OH Vitamin D, Architect system. Abbott, Wiesbaden, Germany). All samples were assayed in two batches to minimize between-run analytic variations. Vitamin D deficiency was defined as serum 25(OH)D < 20 mg/dL.

Overall summary statistics were calculated in terms of means and SD.

Relationship between vitamin D levels and age was evaluated by means of correlation analysis, while relation between vitamin D concentration and gender was studied by means of simple logistic regression, and Point biserial correlation coefficient was calculated to evaluate the relation between vitamin D concentrations and gender.

Serum 25(OH)D levels in the four groups were compared by means of one way-ANOVA followed by a post ad-hoc test (Tukey HSD test)., When patients were redistributed into two groups, Student *t* test was performed.

One way-ANOVA was also performed to exclude significant differences in age and sex distribution among the four groups. A p value equal or less than 0.05 was considered as statistically significant.

## Results

Seventy-eight patients (31 men and 47 women) with an average age of 58.7 years, ranging between 55 to 65 years were included in our study. Distribution of patients in the 4 study groups is summarized in Table 1.

**Table 1:** Characteristics of patients included in the study

	Nr of patients (male/female)	Age (mean ± SD)	Surgical procedure (n)	Serum 25(OH)D (ng/mL) mean ± SD
Group A	23 (5/18)	63.5 ± 0.9	THR (12)	18.52 ± 6.46
			TKR (11)	
Group B	19 (4/15)	59.4 ± 1.5	THR (9)	13.65 ± 3.36
			TKR (10)	
Group C	21 (12/9)	54.1 ± 2.7	SA (12)	16.75 ± 3.58
			OM (9)	
Group D	15(7/8)	63.1 ± 1.3	ACL (4)	16.9 ± 7.24
			Coxarthrosis (5)	
			Fracture (4)	
			Mallet finger (2)	

*THR* Total Hip Replacement, *TKR* Total Knee Replacement, *SA* Septic Arthritis; *OM* Osteomyelitis, *ACL* Anterior Cruciate Ligament Reconstruction

A general 25(OH)D deficiency was observed in the study population, with a mean 25(OH)D value of 16.5 ± 5.42 ng/mL. Age and sex did not significantly affect vitamin D levels, and no significant differences in terms of age and gender were evidenced among the four groups.

Patients with an osteoarticular infection (Groups A and C, *n* = 44, 17 men and 27 women) presented a 25(OH)D serum level of 17.7 ± 5.3 ng/mL, while in patients without infectious diseases (Groups B and D, *n* = 24, 11 men and 23 women) a slightly lower 25(OH)D vitamin concentration was found (15.1 ± 5.6 ng/mL). This difference was statistically significant (*p* < 0.05).

No statistically significant differences were observed when 25(OH)D serum concentration of patients with prosthetic implants was compared with that of patients not carrying joint prostheses (16.3 ± 5.8 ng/mL vs 16.8 ± 5.3 ng/mL, Groups A and B vs Groups C and D). By contrast, when vitamin D serum levels of the four groups were compared by one-way ANOVA, a significant difference in serum 25(OH)D was observed between patients with a PJI (18.5 ± 6.5 ng/mL) and those with an aseptic prosthetic loosening (13.6 ± 9.4) (*p* < 0.05).

## Discussion

The present study was focused on patients undergoing orthopedic surgery and their preoperative 25(OH)D status, related to the eventual presence of a prosthetic failure and/or an infective pathology.

We decided to determine serum 25(OH)D instead of its active form 1,25(OH)_2_D because the former is recommended for determination of vitamin D status, thanks to its longer half-life, and to the fact that it is the most abundant circulating metabolite and the most reliable indicator of vitamin D intake and storage [11, 12].

We recorded a prevalence of 25(OH)D deficiency in 79 % of the study population, higher than that observed by other authors in healthy population, which has been assessed to be about 45 %, [13, 14] even if we used a cut-off value of <20 mg/dL, according to the 14^th^ International Workshop Consensus Conference on vitamin D [15].

The prevalence of 25(OH)D deficiency we have recorded was rather similar to that observed in patients scheduled for arthroplasty [16, 17] and in hospitalized patients, especially those of intensive care unit [18].

Although previous studies proposed vitamin D deficiency as a negative prognostic factor for infections development, [19, 20] we found that patients with a clinically confirmed diagnosis of PJI presented a slight but significant higher serum level of 25(OH)D than those without infections. By contrast, patients without prostheses showed similar 25(OH)D levels in the infected and non infected groups.

Our unexpected results might be due to the fact that previous studies measured vitamin D levels before the onset of infective pathologies, and only severe infections in critical patients presenting co morbidity and hospitalized in intensive care units were considered (bloodstream and low respiratory tract infections), [18, 19] while we considered only patients with clinically confirmed prosthetic or bone infections. Other authors, at last, considered viral infections, which are not comparable to bacterial ones [20].

At our best knowledge, only one study regarding vitamin D level in PJIs patients, considering a study population similar to the one we observed has been performed at the time of manuscript drafting [17]. However, our groups were more homogeneous, and we did not need to apply corrections for physical characteristics and co morbidity status because of the small but strictly selected number of patients, that allowed us to minimize possible confounders.

It is also notable that Maier et al. [17] recorded 25(OH)D values that are overall similar to the ones we observed, but with a different distribution. They found lower 25(OH)D in PJI patients while in our study these patients presented statistically significant higher values respect to the other groups. This difference may be due to the state of the infection; all of our patients, in fact, presented late PJIs, onset at least 12 months after the arthroplasty, while Maier et al. [17] also considered early and delayed infections, which could be associated to a more marked inflammatory reaction in respect to late PJIs that, in turn, has been shown to lead to a decrease in 25(OH)D [21].

Vitamin D has been proven to act as a protective factor against the onset and the severity of a broad type of infections in various studies, [22, 23] while data of this study suggest a different behaviour of vitamin D in PJIs. It could be also hypothesized that the higher levels observed in PJI patients in comparison with patients with aseptic failure of the prosthesis may be the result of the infectious process rather than one of factors predisposing to infection itself. However in this case, higher vitamin D levels should have been observed also in patients with osteomyelitis and aseptic arthritis, that, by contrast, were not found to significantly differ from those observed in not-infected patients. On the other hand, the higher mean age of the study population and the specific stage of PJIs considered are probably the main, but not the only factor determining the results of this study. For these reasons, long prospective studies on how vitamin D levels influences the occurrence of early, delayed and late PJIs could be advisable.

## Conclusions

Our study presents different limitations, first of all the limited study population, and the lack of measurements of serum vitamin D in the follow-up after surgery. Nonetheless, it has been shown that surgery itself can affect 25(OH)D levels, which may decrease after an inflammatory insult [16].

Despite limitations, we evidenced a different scenery in respect to other authors, therefore re-opening the debate on the role of vitamin D status in patients with bone and joint infections.

## Abbreviations

25(OH)D: 25 hydroxyvitamin D
VDR: Vitamin D receptor
PJI: Prosthetic joint infection
THR: Total Hip Replacement
TKR: Total Knee Replacement
SA: Septic arthritis
OM: Osteomyelitis
ACL: Anterior Cruciate Ligament
